# Extracorporeal shock wave therapy in the treatment of primary bone marrow edema syndrome of the knee: a prospective randomised controlled study

**DOI:** 10.1186/s12891-015-0837-2

**Published:** 2015-12-05

**Authors:** Fuqiang Gao, Wei Sun, Zirong Li, Wanshou Guo, Weiguo Wang, Liming Cheng, Debo Yue, Nianfei Zhang, Amanda Savarin

**Affiliations:** Centre for Osteonecrosis and Joint-Preserving & Reconstruction, Beijing Key Laboratory of Arthritic and Rheumatic Diseases, Department of Orthopedic Surgery, China-Japan Friendship Hospital, Beijing, 100029 China; Department of Orthopedic Surgery, China Japan Friendship Hospital, Beijing, 100029 China

**Keywords:** Bone marrow edema syndrome, Extracorporeal shock wave therapy, Knee, Conservative treatment, Magnetic resonance imaging

## Abstract

**Background:**

The aim of this prospective study was to evaluate the effectiveness of extracorporeal shock wave therapy (ESWT) in normalizing the symptoms and imaging features of primary bone marrow edema syndrome (BMES) of the knee.

**Methods:**

This study compared the outcomes of ESWT (Group A) (*n* = 20) and intravenously applied prostacyclin and bisphosphonate (Group B) (*n* = 20) in the treatment of BMES of the knee in our department between 2011 and 2013. The Visual Analog Scale for pain (VAS, 100 mm), the Western Ontario and McMaster University Osteoarthritis Index (WOMAC), the SF-36 scores and MRI scans as well as plain radiographs were obtained before and after therapy between two groups.

**Results:**

Compared with Group B, we found greater improvement in VAS, the WOMAC Osteoarthritis Index and SF-36 score at 1, 3 and 6 months post-treatment in Group A (*P* < 0.05). Furthermore, MRI scans showed a higher incidence of distinct reduction and complete regression of bone marrow edema at 6 months in Group A (95 vs. 65 %; *P* = 0.018). The MRI at 1 year follow-up showed complete regression in all patients in Group A. However, two cases in Group B continued to normalize over the subsequent follow-up period.

**Conclusions:**

ESWT can produce rapid pain relief and functional improvement. It may be an effective, reliable, and non-invasive technique for rapid treatment of BMES of the knee.

**Trial registration:**

Research Registry UIN 528, September 03, 2015.

## Background

Primary bone marrow edema syndrome (BMES) represents a reversible but highly painful increase in interstitial fluid [1, 2]. It is a common finding in MR-imaging of patients with joint pain following largely non-diagnostic or normal radiographs. Although various vascular factors are known to contribute to bone marrow edema (BME), the exact pathogenetic processes are not currently known [3]. The natural time-course for improvement of clinical symptoms and normalization in MRI lasts from 3 to 18 months [4]. BMES has been reported to occur in the knee (BMESK); yet, owing to the small number of reports on this specific entity, little is known about the optimal treatment of patients with this condition [1].

In general, the therapeutic approach to BMESK is based on its suspected etiology and ranges from various symptomatic therapies to core decompression (CD) [1–5]. Non-surgical treatments that have been reported as being beneficial include reduction in weight-bearing load of the joint, analgesic and anti-inflammatory medication, glucocorticosteroids, bisphosphonates, calcium channel blockers and prostaglandin inhibitors (e.g. iloprost) [2–4]. Unfortunately, conservative treatment approaches are unable to relieve symptoms in some cases [1, 5]. Surgical CD, which reduces pain through relief of intraosseous pressure, is usually used as the last resort, particularly as the condition is self-limiting in the majority of patients [1, 5, 6]. BMESK can be associated with a prolonged course of disease and invalidity, non-response to treatment and disease recurrence. Surgical intervention is a costly approach and carries with it the risk of complications including wound infection, hematoma formation, reflex sympathetic dystrophy, and bone fractures associated with bone tunnel drilling [1, 2, 6]. Various treatments have been proposed in an attempt to shorten the natural course of the disease, which is invariably associated with severe and long-lasting disability [5, 7]. However, there is a need for an effective and non-invasive method of treating BMESK.

In musculoskeletal disorders, the effectiveness of extracorporeal shock wave therapy (ESWT) has been widely recognized and recent research supports its use in the treatment of the first stages of avascular osteonecrosis of the proximal femur and in other conditions where bone marrow edema is present [7–9]. The mechanism by which shock wave therapy works is being increasingly broad and in-depth study. It has been shown to activate many cellular processes critical to neovascularization and tissue regeneration. Previous reports have shown that shock wave has also been reported to control inflammatory processes and facilitate bone reparative processes [7–11].

On this basis, we performed a prospective randomised controlled study to evaluate the effectiveness of ESWT in normalizing the symptoms and imaging features of BMESK. We compared 2 therapies, topical ESWT chosen as the observation group versus iloprost and bisphosphonate treatment served as the control group. We hypothesized that topical ESWT would result in rapid pain relief and functional improvement without substantial complications.

## Methods

This prospective randomised controlled study was approved by the Ethics Committee of China-Japan Friendship Hospital (China-Japan Friendship Hospital drug / device clinical trials Ethics Committee). Written consents were provided by the patients to be stored in the hospital database and be used for clinical research.

### Clinical data

In this single center study, 40 consecutive patients with MRI-confirmed primary BMESK were prospectively matched by age and diagnosis from June 2011 to May 2013 (Table 1). All patients provided written informed consent to participation in this prospective trial, and the study was approved by the Scientific Review Board of our institution. Using computer-generated random assignment concealment with sealed envelopes, patients were allocated to receive ESWT (Group A) (*n* = 20) or alendronate sodium tablets (70 mg po qw; Merck & Co., Inc.; Peking) and alprostadil (10 μg ivgtt qd; Peking Tide Pharmaceutical Co., Ltd.; Peking) (Group B) (*n* = 20). Exclusion criteria were BME with any finding of avascular necrosis (demarcation) or advanced osteoarthritis (Ahlbäck grade 3 or 4). Patients who had received any previous treatment were also excluded, along with those who had contraindications for ESWT [8]. The average time-period between the onset of symptoms and the beginning of treatment was 3.8 weeks (range 2–8 weeks). All patients were mobilized with partial weight-bearing and walking aids for 6 weeks and analgesics on demand with restrictions for impact sports such as sprinting or jumping. The groups were examined clinically and evaluated with relevant scoring systems by a single examiner: the Visual Analog Scale for pain (VAS, 100 mm), the Western Ontario and McMaster University (WOMAC) Osteoarthritis Index and the SF-36 scores were assessed before treatment (t0), at 1 months (t1), 3 months (t2), 6 months (t3) and 1 year (t4) post-treatment. MRI scans as well as plain radiographs were obtained before, and 6 months and 1 year after, therapy. The mean follow-up period was 13 (12–18) months. An experienced radiologist evaluated the area of edema on one slide with the most obvious edema of the resulting MRI films with the same fluid sensitive sequence using the PACS software (Kodak version 11.0, MA, USA) to verify whether the edema lesion showed unchanged, reduced or regressed completely.

**Table 1:** Patients characteristics

Characteristics	Group A (*n* = 20)	Group B (*n* = 20)
Female, *n* (%)	9 (45)	11 (55)
Age, years	41.6 ± 9.7	45.1 ± 8.9
Height, m	1.65 ± 0.21	1.68 ± 0.32
Weight, kg	64.2 ± 6.3	67.4 ± 8.6
BMI, kg/m^2^	25.5 ± 3.7	24.9 ± 5.4
IBST, weeks	2.9 ± 2.7	3.2 ± 1.9
Duration of follow-up, months	12.1 ± 2.5	13.0 ± 1.8

Note: *BMI* Body Mass Index, *IBST* Interval between the onset of symptoms and the beginning of treatment

### Shock wave treatment

The shock wave treatment was applied using an Electromagnetic Shock Wave Emitter (Dornier Compact DELTA II; Germany), with a penetration depth of between 0 and 150 mm and a focus diameter of 4 mm. Shock waves were focused around (on the margins of) the femoral head under radiographic guidance. The treatment area was prepared with a coupling gel to minimize the loss of shock wave energy at the interface between the head of the device and the skin. In Group A, patients were subjected to high-energy ESWT [12, 13], and the parameters are prepared and used as follows: number of levels, 3–4; at a high energy flux density (EFD) of > 0.44 mJ/mm^2^ (level 3); 3000–4000 impulses at a frequency of 2–3 Hz. Each patient underwent two therapy sessions (the time interval between successive procedures was 1 week). The number of the frequency selected depends on the patient’s condition.

### Statistical Analysis

All data analyses were performed using SPSS version 16.0.0 software (SPSS; Chicago, IL, USA). The means and standard deviations (SD) were calculated for all patients, and 95 % confidence intervals (CIs) were determined. For within-group comparisons, we performed a paired *t*-test. A probability (*P*) value < 0.05 was considered to be of statistical significance.

## Results

**Fig. 1:**
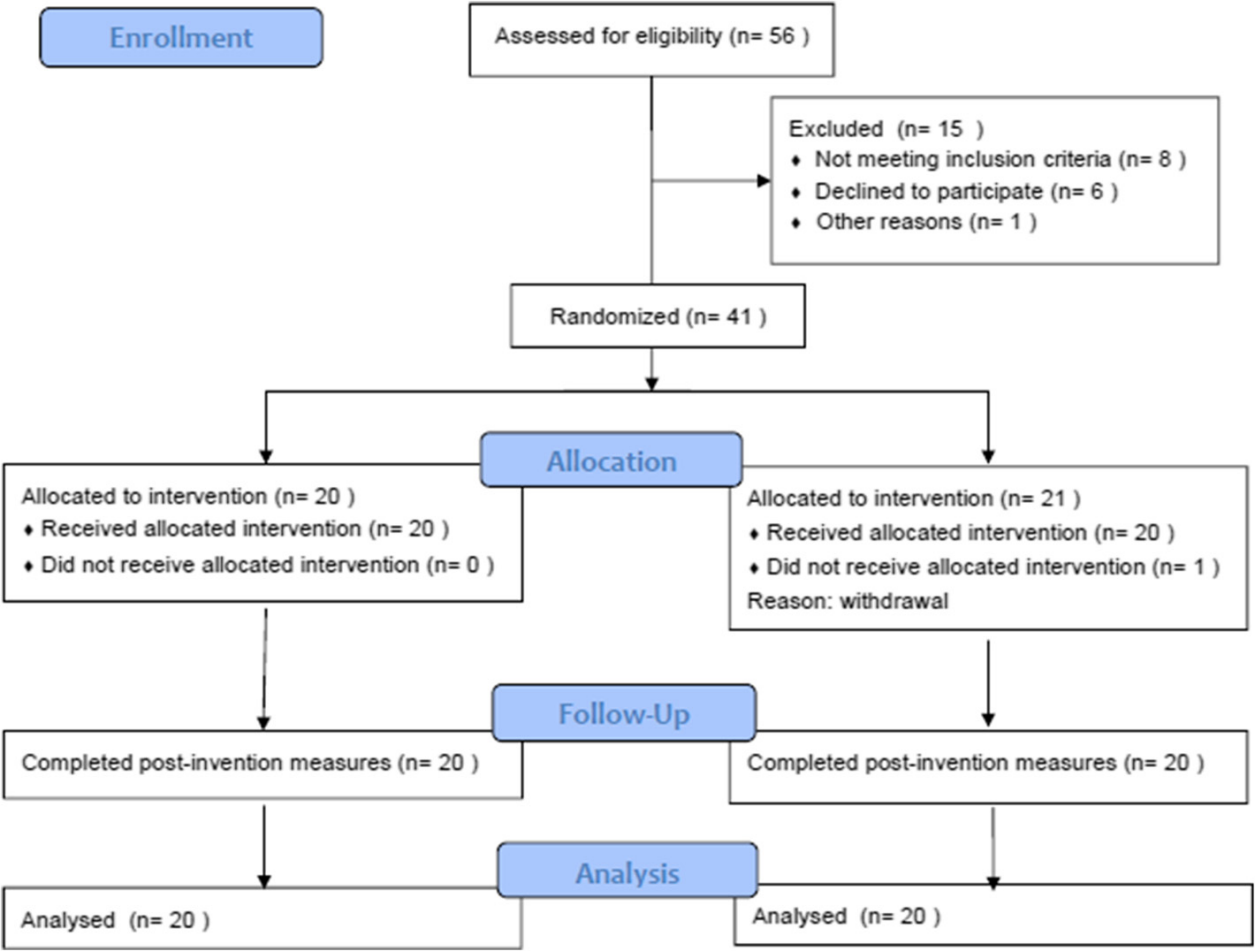
The CONSORT flowchart in the trial

From June 2011 through May 2013, 56 patients with MRI-confirmed BMESK with a single group of surgeons were screened and assessed for eligibility, as 9 patients were eliminated by exclusion criteria. 6 patients were excluded from the trial due to patient decline of enrollment. A total of 41 patients were randomized to receive the study treatment using a block randomization technique. After randomization, one patient withdrew consent prior to the study, thus resulting in 40 patients (group A: 20 patients for ESWT; group B: 20 patients for drugs) that were included in the statistical analysis of our trial (Fig. 1).

### Clinical results

**Fig. 2:**
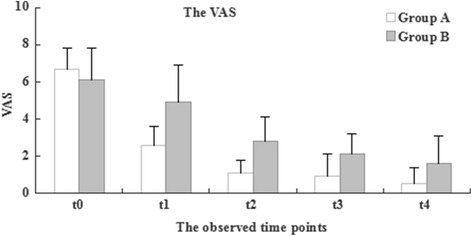
The development of the VAS during therapeutical intervention between two Groups

**Fig. 3:**
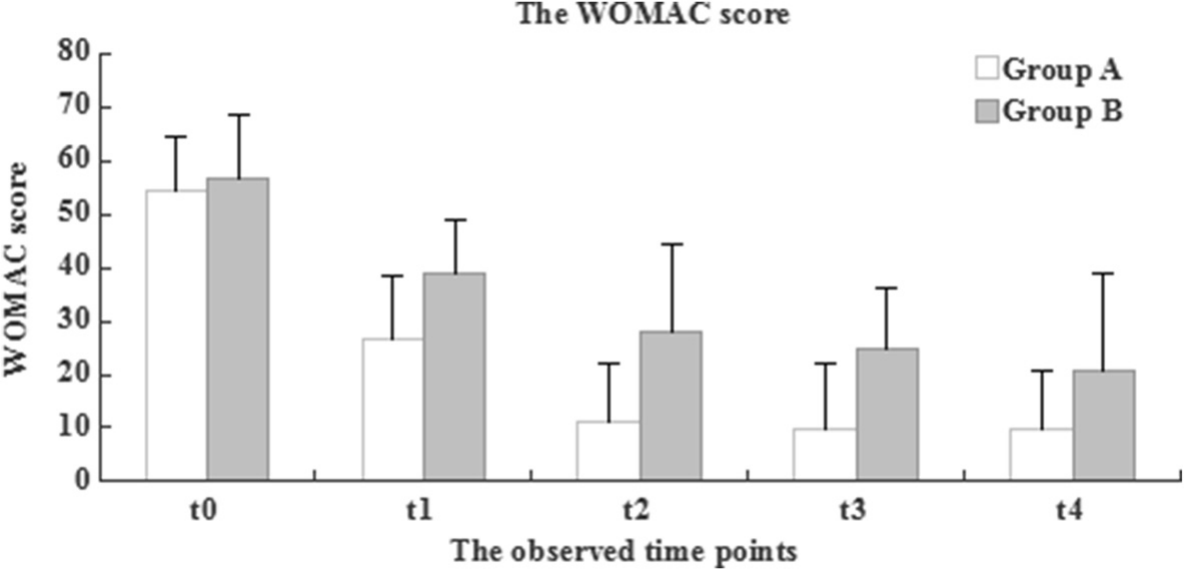
The development of the WOMAC score during therapeutical intervention between two Groups

**Fig. 4:**
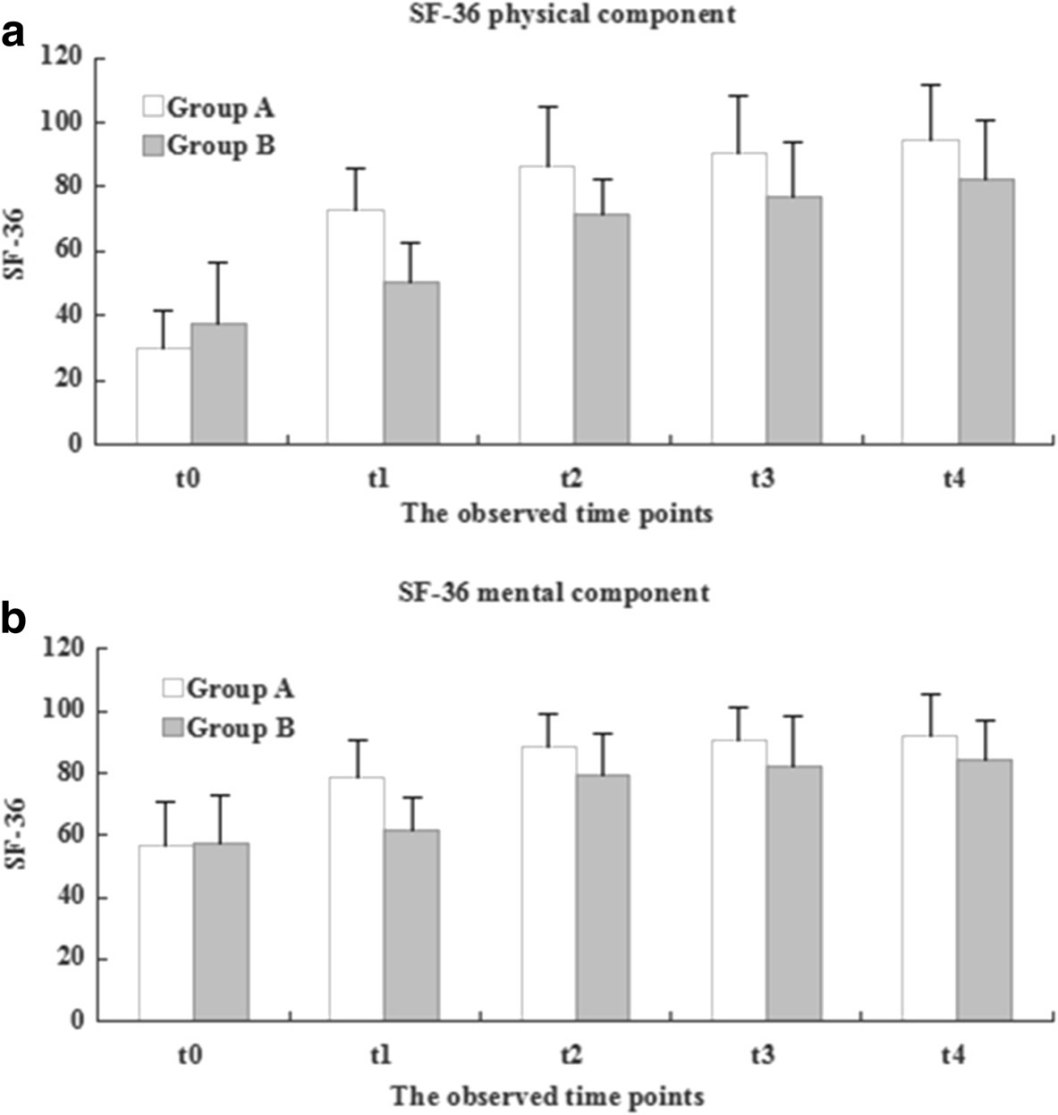
The development of the SF-36 score during therapeutical intervention between two Groups (**a**, physical component; **b**, mental component)

Compared with Group B, all patients in Group A showed a greater and earlier improvement in VAS, WOMAC Osteoarthritis Index and SF-36 score at t1–3 (1, 3 and 6 months post-treatment) after therapeutic intervention (*P* < 0.05), and almost all patients in Group A continued to improve over the follow-up period (Figs. 2, 3 and 4). Significant improvement in the VAS was observed in Group A, from 6.7 ± 1.1 points to 2.6 ± 1.1 points at 1 month (t1) and to 1.1 ± 0.7 points at 3 months (t2) after therapeutic intervention (*P* < 0.001) (Fig. 2). Gradual improvement in the VAS was shown in Group B, from 6.1 ± 1.7 points to 4.9 ± 2.0 points at 1 month (t1) and to 2.8 ± 1.3 points at 3 months (t2) after therapeutic intervention (*P* < 0.05) (Fig. 2). The mean improvement between t2 and t3 and between t3 and t4 in both groups was not statistically significant.

Significant improvements in the WOMAC Osteoarthritis Index were observed in both groups (Fig. 3). Group A improved from 54.3 ± 10.1 points to 26.6 ± 12.0 points at 1 month (t1), to 10.9 ± 11.1 points at 3 month (t2), to 9.8 ± 12.3 points at 6 months (t3) and to 9.7 ± 11.0 points at 1 year (t4) after ESWT (*P* < 0.01) (Fig. 3). Group B showed significant improvement, from 56.9 ± 11.5 points to 38.7 ± 10.1 points at 1 month (t1), to 27.8 ± 16.7 points at 3 months (t2), to 24.6 ± 11.3 points at 6 months (t3) and to 20.9 ± 17.8 points at 1 year (t4) after drug therapy (*P* < 0.05) (Fig. 3). There was a trend towards a greater improvement in Group A than in Group B; this effect was statistically significant (*P* < 0.01).

The SF-36 score improved significantly in both groups at t1 (1 months) (Fig. 4). There was a statistically significant difference in the physical component of the SF-36 score between the groups at 3 months (*P* < 0.05) (Fig. 4a). However, there was no significant difference in the mental component of the SF-36 score between the groups at 3 months (*P* > 0.05) (Fig. 4b). Comparison of the results within and between the two groups revealed that there were no significant differences in the VAS, WOMAC Osteoarthritis Index and SF-36 score at 1 year post-intervention (*P* < 0.05).

### Radiological results

The MRI findings demonstrated the progressive regression of the BME. MRI scans of both groups showed that the patients in Group A had a higher incidence of distinct reduction and complete regression of BME at 6 months (95 vs. 65 %; *P* = 0.018). The MRI at 6 months follow-up in Group A showed a reduction in BME in 35 % (7/20) of all patients and complete regression in 65 % (13/20). In Group B, there was a reduction in BME in 40 % (8/20) of all patients and complete regression in 25 % (5/20). However, the MRI at 1 year follow-up showed complete regression in all patients in Group A (100 %) and most patients in Group B (90 %); two cases in Group B continued to normalize over the subsequent follow-up period (18 months).

### Side-effects

Only minor complications occurred after ESWT, such as transient soft tissue swelling or minor bruising. No other adverse effects were noted. No clinically detectable neuromuscular, systemic, or device-related adverse effects were observed in the ESWT group. Following alprostadil administration, headache was reported in three patients and a facial rash in two patients; these symptoms occurred during the first 1 h of infusion. No adverse events were detected with alendronate.

### Case report

#### Case 1

**Fig. 5:**
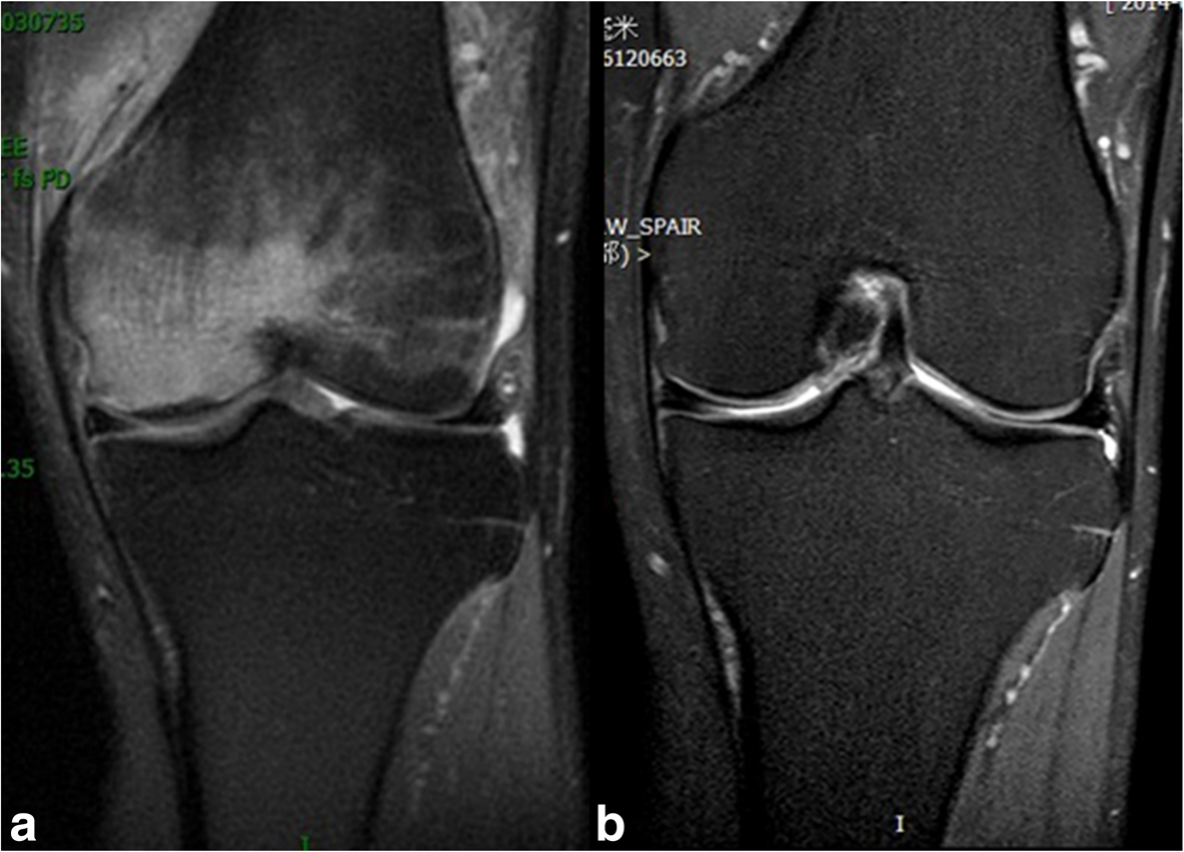
Pre- (**a**) and 6 months posttreatment (**b**) T2-weighted images showing the normalization of a large bone marrow edema within the medial femoral condyle of the left knee, in a 62-year old male patient. (Note: The patient consented to publish the specific information.)

Bone Marrow Edema Syndrome (BMES) of the left knee was diagnosed in a 62-year-old, male professor. ESWT rapidly produced positive effects with regard to both pain and BME. The VAS score dropped from 8 points preoperatively to 2 points at 1 months post-treatment. Symptoms of pain were significantly alleviated. In addition to improvements in the WOMAC Osteoarthritis Index, SF-36 score and VAS, MRI showed a significant reduction in edema between the pre-treatment (Fig. 5a) and 6-months post-treatment (Fig. 5b) time-points. The patient has provided consent to publish the information contained in this case report, as well as Fig. 5 and the accompanying legend.

## Discussion

Our study shows that ESWT can also relieve a great deal of discomfort for BMESK patients. Furthermore, the mean VAS showed a dramatic improvement from pre-treatment values at all follow-up time-points in ESWT patients, especially at t1 (1 month). The clinical improvement in WOMAC Osteoarthritis Index and SF-36 scores observed following ESWT was obvious in most patients at 1 month post-treatment (*P* < 0.05). These scores improved significantly earlier than those in the drug control group (*P* < 0.05), in which improvements were observed mainly at 3 months post-intervention. At this point, all patients had already regained a significant level of autonomy in their daily lives with a marked reduction in pain, which correlated with the progressive normalization of MRI features. Although the final outcomes of the various conservative treatments currently used are similar to those observed in some studies of ESWT, it is important to note that ESWT is a simple, non-invasive treatment that does not require the administration of pharmacological drugs, thus avoiding the reported potential side-effects [8]. Our treatment protocol required only two therapy sessions as opposed to the time-consuming extended treatments such as pharmacological drugs.

Primary bone marrow edema syndrome is a rare, but underdiagnosed source of pain which mainly occurs around joints of the lower extremities [2, 5, 14]. There is still debate regarding the pathogenesis and implications of BME, and this is reflected in the lack of a gold standard in the treatment of this condition. Because of the reversibility of BME, conservative treatment has been recommended, including reduction of weight-bearing load, analgesic and anti-inflammatory medication and physiotherapy [5, 6, 11, 14, 15]. Reports of the use of bisphosphonates relate predominantly to their role in the treatment of BMES. Intravenous prostacyclin and bisphosphonate can be used to achieve a reduction in BME, with a considerable improvement in the accompanying symptoms [2]. Prostacyclin improves tissue blood supply in a variety of situations through multiple mechanisms, including vasodilatation and inhibition of platelet aggregation [2, 5]. Pain relief and rapid regression of BME is attributed to the action of prostacyclin in dilating vessels and reducing capillary permeability [14]. Bisphosphonates have been shown to improve bone density in a variety of conditions [2]. Unfortunately, conservative treatment approaches take too long time or are unable to relieve symptoms in some cases [1, 5]. In attempting to shorten the clinical course BMES, which is invariably associated with severe and long-lasting disability, various treatments have been proposed [5, 7, 14]. Core decompression has been reported as the standard surgical treatment of recurrent or persistent painful BMES, particularly of the hip, and improves symptoms of pain through relief of intraosseous pressure [1, 2, 5, 7, 16, 17]. However, surgery is costly and associated with risks [2, 5, 6]. A consensus is required regarding the importance of an early treatment to relieve pain and to avoid weakening the bone trabeculae, which could potentially lead to a collapse or fracture of the subchondral bone [8]. Some consider that surgery is too invasive for a self-limiting disease with a variable clinical course [2, 8, 15].

ESWT has been shown to be effective in treating many orthopedic disorders, including osteonecrosis [1, 9, 18]. Clinical trials have also highlighted the effectiveness of ESWT in treating the early stages of avascular necrosis, reducing bone edema and pain [8, 18]. There are currently few reports addressing the use of ESWT in BMES of the hip [7, 8, 11]. However, the exact mechanism by which ESWT operates remains relatively unknown. Tischer et al. demonstrated the amount of new bone formation is directly dependent on the applied EFD [10]. Too low or too high defined energy dose for shock wave applications is disadvantageous to formation of new bone. So it is very important to select an appropriate EFD, and it can improve the efficacy of shock wave and minimize topical side effects [10]. The close anatomical and functional links between vascular elements, marrow stromal and active bone cells may explain the positive effects of ESWT on bone metabolism [8]. ESWT, as one of the most frequently used physical therapies, seems to be able to control inflammatory processes and to facilitate bone reparative processes as well as to activate many cellular processes critical to neovascularization and tissue regeneration [7–11]. Some animal studies have demonstrated positive results in the application of ESWT on osteonecrosis with a better induction of tissue ingrowth and neovascularization; it is associated with increased expression of angiogenic growth factors, including BMP-2, vessel endothelial growth factor (VEGF), endothelial nitric oxide synthase (eNOS), and proliferating cell nuclear antigen (PCNA), and it promotes cell proliferation and osteogenesis [19–21]. BMP-2 is a key mediator of bone development and repair through its capacity to mobilize osteoprogenitor cells. Increased expression of BMP-2 has been identified in femoral heads treated with ESWT, thereby promoting osteoblastic differentiation processes and resulting in bone formation [22]. VEGF, as a specific mitogenic factor for vascular endothelial cells, may be involved in the mechanism of the positive effects of ESWT. It stimulates the proliferation of endothelial cells, promotes neovascularization, and increases vascular permeability [23]. Finally, eNOS promotes neovascularization [18]. It is reasonable to speculate that neovascularization plays a role in the improvement in the blood supply to the femoral head and may promote bone regeneration in cases of BMES [24, 25]. Early studies indicate that a similar effect to that of vasoactive drugs can be obtained with ESWT [9, 17]. The neo-angiogenetic effect of ESWT appears to reduce the time to symptom remission. ESWT showed significantly better clinical results and BME regression rates in MR-imaging compared to conservative treatment in combination with partial weight-bearing in the treatment of BMESK and shortens the natural course of the disease. Besides, ESWT might have the potential to avoid the need for surgical intervention according to previous studies [26, 27].

The pathophysiologic mechanism that is responsible for the dramatic pain reduction in all BMES patients following ESWT, is still unclear. ESWT for the management of BMES is easy to perform in a clinical setting and does not carry risk for the patients. Our results indicate that ESWT of BMES with increased bone turnover reduces pain by eliminating the BME and shortens the natural course of the “self-limiting disease”. The high regression rate of BME with no bone osteonecrosis present at the follow-up MRI in this study also supports the hypothesis that BMES is a distinct clinical entity, rather than an early form of bone osteonecrosis [14, 28, 29].

This study was conducted according to the CONSORT guidelines [30] with a rigorous study design, a clinically feasible intervention and good adherence to the programme. There are limitations associated with this study. The number of patients in the study was relatively small, and the follow-up time was relatively short, but similar to prior studies on this subject. The functional improvement in the knee was assessed subjectively using the VAS and functional scores, but no objective measures were utilized. However, the results of this study warrant confirmation in larger trials with longer follow-up periods although these studies are complicated by the off-label-use of ESWT. These patients with ESWT had not gotten any iloprost and bisphosphonate. However, we will consider combining topical ESWT with iloprost and bisphosphonate in the treatment of BMESK in future studies.

## Conclusions

In summary, ESWT is an effective, reliable, and non-invasive technique for rapid treatment of BMESK, followed by a progressive normalization of the MRI appearance. ESWT represents an innovative technology applicable to orthopedics, although further development is required. Further exploration of its mechanisms and prospects would be worthwhile, as it has the potential to resolve the suffering of BMESK patients rapidly and effectively.

## Abbreviations

ESWT: Extracorporeal shock wave therapy
VAS: Visual Analog Scale for pain
WOMAC: Western Ontario and McMaster Universities Osteoarthritis Index
BMES: Bone marrow edema syndrome
MRI: Magnetic resonance imaging
CD: Core decompression
EFD: Energy Flux Density
VEGF: Vessel endothelial growth factor
eNOS: Endothelial nitric oxide synthase
PCNA: Proliferating cell nuclear antigen
